# Unintended pregnancy and its correlates among currently pregnant women in the Kwango District, Democratic Republic of the Congo

**DOI:** 10.1186/s12978-016-0195-z

**Published:** 2016-06-16

**Authors:** Sarita Dhakal, Jin Sung Song, Dong Eun Shin, Tae Ho Lee, Ae Young So, Eun Woo Nam

**Affiliations:** Yonsei Global Health Center, Yonsei University, Wonju, Gangwon-do Korea; Department of Health Administration, Graduate School, Yonsei University, Wonju, Korea; Institite for Poverty Alleviation and International Development (IPAID), Yonsei University, Wonju, Korea; Department of Nursing, Gangneung-Wonju National University, Wonju, Korea

**Keywords:** Unintended pregnancy, Reproductive age women, DR Congo

## Abstract

**Background:**

Unintended pregnancy is an important reproductive health problem in both developed and developing countries and is most prominent in low-middle income countries. In the Democratic Republic of the Congo, the total fertility rate is high at 5.9 births per women, and a mother’s probabily of dying at an age between 15–49 years is also high (53 %). Women with unintended pregnancies are less likely to utilize available necessary services for their own health and the health of their children. Therefore, unintended pregnancy is a crucial factor of maternal health in the Democratic Republic of the Congo. This study aims to identify the prevalence of unintended pregnancy and its associated factors in the Democratic Republic of the Congo.

**Methods:**

Data were collected from June 20 to 29, 2014 among women aged 15–49 years who had children younger than 5 years old. The women were from a representative sample of 602 households. Multiple logistic regression analysis was performed to evaluate the associations between the dependent variable and the explanatory variables.

**Results:**

Unintended pregnancy was reported in 51.4 % of the respondents. Multivariate logistic regression showed an association between education status (AOR, 3.4; CI, 1.21–9.90) and age of the last child (AOR, 5.17; CI, 1.23–21.70) with an unintended pregnancy. Unintended pregnancies were low among women who owner a cell phone (AOR, 0.18; CI, 0.47–0.73) and those who were aware of family planning method (AOR 0.20; CI, 0.06–0.60).

**Conclusion:**

The unintended pregnancy rate high and was significantly associated with female education, previous use of family planning methods, ownership of cell phone, and age of the last child. Maternal health interventions should focus on increasing family planning service utilization, awareness of family planning, and access to communication and income.

## Background

The Democratic Republic of the Congo (DRC) faced four decades of war and conflict, which had many repercussions on societal and economic development. Approximately 40 % of the population, nearly 70 million inhabitants, lives in urban areas according to the latest NSI (National Statistics Institute) estimate [1]. Despite an impressive economic growth rate and a reduction in the poverty rate from 71 % to 63 % between 2005 and 2012, the poverty rate remains high [2]. The country ranks second to last on the Human Development Index (186 out of 187 countries) [3] and its per capita income is very low. More than 70 % of Congolese citizens earn less than $ 1 a day, which is among the lowest in the world. There are 200–250 ethnic groups each with a unique language or dialect and customs [4].

An unintended pregnancy is one which is either mistimed (i.e., occurred earlier than desired) or unwanted (i.e., occurred when no children or no more children were desired) [5]. It is a complex issue not only due to individual behavior, but is also aided by also public policy and institutional practices [6].

According to the Demographic Health Survey of the DRC, the maternal mortality ratio was 846/100,000 live births, and women of reproductive age are at have a1 in 18 risk of dying from maternal causes [7]. Unintended pregnancies continue to be a problem in developing and developed countries [8]. One of the consequences of unintended pregnancy is an increase in the rate of induced abortions, which is more problematic in countries where abortion is illegal [9, 10]. Because DRC is a country where abortion is not legal access to safe abortion services is very difficult. The country completely prohibits abortion, so there is no legal exception to save the life the mother [11, 12]. Unintended pregnancy is an important public health issue in developed and developing countries due to negative associations with the social and health outcomes of both the mother and child [13–15]. Compared to intended pregnancies, mothers with an unintended pregnancy utilize less and delay prenatal and postnatal care [16, 17]; breastfeed for a shorter duration; have poorer personal hygiene; and have higher rates of risky behavior such as smoking, drinking alcohol, and drug abuse during pregnancy. Children born after an unintended pregnancy are also more likely to have poor nutrition, a lower birth weight, incomplete vaccinations, and a higher incidence of illness compared to those of intended pregnancies [18, 19].

The purpose of this study is to examine the current incidence of unintended pregnancies among women of reproductive age in Kenge and Boko, DRC, and to explore the factors associated with unintended pregnancies.

## Method

This study utilizes baseline household survey data for the Maternal and Child Health Project of the DRC. Representative sample is calculated by using “Raosoft” [20]. Considering total population (309,468), margin of error of 5 % and the response distribution of 50 %, the sample size will be 384. So, 602 household were selected and in these households, 754 respondents (women of reproductive age) were selected. Out of all the respondents, currently pregnant (105) were selected. A multistage sampling technique was used to select 754 respondents. At each health zone (Kenge and Boko), 24 health areas were selected. At each health area, villages were grouped into three strata: near villages (within 5 km from the health center) average villages (located between 5 and 10 km from the health center) and remote villages (located more than 10 km from the health center). The study was conducted in three villages including one in each stratum. The number of households per village was proportional to the population size of each stratum. In each village, households were selected random walk from a single entry point of the village. This entry point was selected by simple random sampling. Data representing women aged 15–49 years with children younger than 5 years old were collected from a representative sample of 602 households through collaboration with the Korea International Cooperation Agency (KOICA) from June 20 to 29, 2014. Among 754 respondents, only currently pregnant women (105) were selected for this study. The collected information included: household information on socio-demographics, water and sanitation, handwashing practices, access to mass media and use of information/communication technology, recognition of Maternal and Child Health (MCH) services, fertility, birth history, maternal and newborn health, HIV/AIDS, self-perceived health, post-natal health checks and contraceptive use. In every respondent’s residence, a face-to-face interview to complete a questionnaire was conducted by team of School of Public Health, Kinshasa University and Yonsei Global Health Center, Korea. Each interview lasted approximately one hour. Ethical approval for this study was obtained from the Institutional Review Board of Wonju Campus, Yonsei University (1041849–201406-BM-027–01) and the local DRC government. Informed consent was obtained from individual participants.

**Fig. 1:**
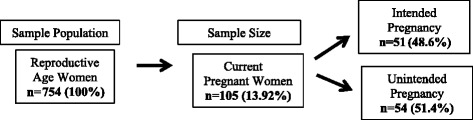
Study sample population

Statistical analysis including the Chi-square test and logistic regression analysis was performed using SPSS (version 21.0). The main outcome (variable) of this study is the intention of the pregnancy. Currently pregnant women were asked if their pregnancy was intended or unintended. The variable was coded as “1” if the pregnancy was unintended and as “0” if it was intended. A breakdown of the study sample population is shown in Fig. 1.

## Results

### Characteristics of the study subjects

A total of 754 women of reproductive age from 602 households were considered for this study. The general characteristics and pregnancy intentions of the study subjects are presented in Table 1. The majority of households (75 %) were categorized as having a low monthly family income of 0.5 to 53 US dollars per month. Education status was divided into two categories: 60.2 were uneducated and 38.8 % were educated. A total of 52.1 % respondents reported that their health facility is located within 5 km from their home and the remaining 47.9 % reported that the distance to the nearest health facility was more than 5 km from their residence. An average of 1.2 women of reproductive age was living in each household. Out of the 754 reproductive age women in the sampled household 13.92 % were currently pregnant; among these pregnancies, 51.4 % were unintended and the remaining 48.6 % (*n* = 51) were intended pregnancy. The highest proportion of unintended pregnancies (*n* = 31, 43.4 %) was observed in the 25–35 age group, which accounted for 60.4 % of the total unintended pregnancies. Additionally, 63 (60 %) pregnant women were educated, while 42 (40 %) were uneducated. The unintended pregnancy rate among the educated women was 60.3 %, which was 1.6 times higher than in uneducated women. The unintended pregnancy rate was lower in the women who were aware of family planning or used contraceptives.

**Table 1:** Characteristics of the study population (n: 105)

Variables	Unintended pregnancy [n (%)]	Intended pregnancy [n (%)]	Total [n (%)]	*X* ^2^	*P*-value
Reproductive age women			745		
Pregnancy status	54 (51.4)	51 (48.6)	105(13.92)	-	-
Age (in years)					
15–24	12 (41.4)	17 (58.6)	29 (30.2)	1.975	0.373
25–35	31 (53.4)	27 (46.6)	58 (60.4)		
35–49	3 (33.3)	6 (66.7)	9 (9.4)		
Income level (US$)					
Less than $53	39 (50.6)	38 (49.4)	77 (73.3)	5.857	0.053
$54–106	6 (35.3)	11 (64.7)	17 (16.9)		
More than $107	9 (81.8)	2 (18.2)	11 (10.4)		
Educational status					
Educated	38 (60.3)	25 (39.7)	63 (60.0)	4.982	0.026
Uneducated	16 (38.1)	26 (61.9)	42 (40.0)		
Distance to health facility					
Up to 5 km	28 (54.9)	23 (45.1)	51 (48.6)	0.479	0.489
More than 5 km	26 (48.1)	28 (51.9)	54 (48.6)		
Age of the last child					
1–2 years	20 (62.5)	12 (37.5)	32 (31.1)	6.320	0.042
3 years	25 (55.6)	20 (44.4)	45 (43.7)		
More than 3 years	8 (30.8)	18 (69.2)	26 (25.2)		
Family size					
1–4	21 (39.6)	32 (60.4)	53 (50.5)	5.971	0.015
5 or more	33 (63.5)	19 (36.5)	52 (49.5)		
Ownership of a cell phone					
Yes	7 (29.2)	17 (70.8)	24 (22.9)	6.172	0.013
No	47 (58.0)	34 (42.0)	81 (77.1)		

### Contraceptive use

Table 2 shows the results regarding contraceptive use. A total of 44.8 % of subjects reported that they usually use a family planning (FP) method. Of note, the use of a family planning method is gradually increasing in DRC. In this study, 49.5 % of women reported recent used family planning (FP) method (before being pregnant), whereas 50.5 % reported not utilizing any family planning method. In reproductive age women using FP method, 43.6 % were utilizing temporary methods and 56.4 % were utilizing natural methods.

**Table 2:** Use of family planning methods

Variables	Unintended pregnancy [n (%)]	Intended pregnancy [n (%)]	Total [n (%)]
Ever used family planning			
Yes	15 (34.9)	28 (65.1)	43 (44.8)
No	34 (64.2)	19 (35.8)	53 (55.2)
Recent user of family planning			
Yes	15 (30.0)	35 (70.0)	50 (49.5)
No	36 (70.6)	15 (29.4)	51(50.5)
Preferred family planning method			
Temporary	10 (41.7)	14 (58.3)	24 (43.6)
Natural	10 (32.3)	21 (67.7)	31(56.4)

Natural methods: lactational amenorrhea, periodic abstinence/rhythm, withdrawal

### Factors related to unintended pregnancy

Bivariate analysis including the unadjusted odds ratio (OR) are shown in Table 3. Only four factors were associated with unintended pregnancies: education status (AOR, 3.4; CI, 1.21–9.90), age of the last child (AOR, 5.17; CI, 1.23–21.70), ownership of a cell phone (AOR, 0.18; CI, 0.47–0.73), and ever used of a family planning method (AOR, 0.20; CI, 0.06–0.60).

**Table 3:** Factors related to unintended pregnancy

Variables	Crude OR (95%CI)	P value	Adjusted^a^ OR (95%CI)	*P* value
Age (in years)				
15–24	0.70 (0.14–3.40)	0.667		
25–35	0.43 (0.09–1.91)	0.271		
36–49	1			
Income				
Less than $53	0.22(0.04–1.12)	0.069	0.17(0.025–1.24)	0.082
$54–107	0.12(0.02–0.75)	0.024	0.12(0.13–1.19)	0.125
More than $54	1		1	
Education status				
Educated	2.47 (1.10–5.50)	0.027	3.4 (1.21–9.90)	0.021
Uneducated	1			
Distance to health facility				
Up to 5 km	1.31 (0.60–2.82)	0.489		
More than 5 km	1			
Age of the last child				
1–2 years	3.75 (1.25–11.24)	0.018	5.17 (1.23–21.70)	0.025
3 years	2.81 (1.01–7.79)	0.047	2.92 (0.78–10.90)	0.11
More than 3 years	1		1	
Family size				
1–4	0.37 (0.17–0.83)	0.016	1.82 (0.66–5.03)	0.24
5 and more	1		1	
Ownership of a cell phone				
Yes	0.29 (0.11–0.79)	0.016	0.18 (0.47–0.73)	0.016
No	1		1	
Ever used family planning method				
Yes	0.29 (0.19–0.69)	0.005	0.20 (0.06–0.60)	0.004
No	1		1	

^a^Variables entered were income status, education status, age of the last child, family size, ownership of a cell phone, and ever use of a family planning method. Hosmer and Lemeshow value: Chi-square = 4.961, *P* = 0.762, and Nagelkerke R = 0.419

## Discussion

This study aimed to identify the prevalence and related factors of unintended pregnancy in the DRC. More than half (51.4 %) of the pregnancies in the country based on this study sample were unintended. This rate is higher in comparison to rates reported for other developing countries in previous studies. For example, unintended pregnancy rate were 28 in Nigeria [21], 28.2 in Iran [22], 14.3 in Senegal [23], 34 in Ethiopia [24], 41 in Nepal [25], and 54.1 % in Tanzania [26]. In Nigeria, married women were less likely to have an unintended pregnancy, however women with 3 or more children, who were Catholic and Muslim, and those with a low socioeconomic status had a higher probability of having an unintended pregnancy. An unintended pregnancy in Iran was positively associated with women’s age and negatively associated with contraceptive use. Studies in Senegal demonstrated that low socioeconomic status less involvement of married women on joint decision making with their partner (i.e., management of financial resources) and use of family planning are associated with a higher prevalence of unintended pregnancies. Similar results were found in a study conducted in Ethiopia; a history of previous unintended pregnancy, a husband who did not want to limit the family size, a desire for at least two children, 3–4 pregnancies, and parity of 5 or more were factors significantly associated with unintended pregnancy. In Tanzania, women aged less than 20 years who did not want to get pregnant and who lacked inter-partner communication about FP had increased risk of mistimed pregnancy, and the unintended pregnancy rate was also high among multigravida women.

In the present study, bivariate analysis showed that variables such as education status, age of the last child, family size, ownership of a cell phone, and past use of contraception were important factors in the likelihood of an unintended pregnancy. Multivariate analysis supported some of the findings of the bivariate analysis. In multivariate analysis, education status, age of the last child, ownership of a cell phone, and ever used of family planning method were found to have a statistically significant influence on unintended pregnancy.

Educated women in the present study had a higher probability of having an unintended pregnancy in comparison to uneducated women. In contrast, education was not a significant factor of unintended pregnancy in Nepal [25]. However, a discussion document from a global consultation on Developing an Education Sector Response to Early and Unintended Pregnancy showed that education plays a crucial role in efforts to decrease unintended pregnancy rates [26]. Because the fertility rate is very high (5.9) in the DRC, educated women may consider their pregnancies as unintended in compared to uneducated women because educated women want to limit their family size. A similar result is presented in the Sarmad study which found that a higher education level was associated with a smaller found number of children [27] Age of the last child was also found to be related to unintended pregnancy (AOR, 5.17; CI, 1.23–21.70); however, the confidence interval has a wide gap because of the small sample size [28]. Comparison to mothers with children 3 years and older, the probability of unintended pregnancy was higher among mothers who had younger children. This finding suggests that women in the study area desire a longer birth interval. A similar result was observed in a study conducted in the Jimma zone of Ethiopia, which showed that a pregnancy was more likely to be unintended in women who had a previous birth interval of less than 24 months (OR, 1.78; 95 % CI,1.19–2.69) [29]. In the present study, women who had access to a cell phone were less likely to report an unintended pregnancy. Ownership of a cell phone is a form of access to property and provides a means of communication with family and friends, indicating women empowerment. In this study, we assessed only the overall household income and not the monthly income of the women themselves. The household income showed a significant association with the type of pregnancy, however, the income status was not significantly associated with unintended pregnancy in the multivariate analysis. Previous studies have indicated income as one of the factors influencing utilization services including FP. In fact, FP services are usually provided at free of cost in most developing countries including Congo [30]. Based on these results, women who had ever used contraceptives had a lower likelihood of an unintended pregnancy. Similarly, a study conducted in India by Dixit et al. [31] indicated that unintended pregnancy is more common among those who have never used FP method. Additionally, family size was a factor influencing unintended pregnancy. A pregnant women with 4 or fewer family members is less likely to report that her unintended pregnancy is unintended as comparison to those with a larger family size. This is good indication that women want to limit their family size [32].

Many previous studies have shown that the age of the woman [23], distance to health facilities [33], and religion [34] are significantly associated with unintended pregnancy. However, in the present study, statistically significant associations were not found between type of pregnancy and age group, religion, or distance to health facilities.

A lack of contraception is a leading cause of unintended pregnancy. Every year, more than 120 million couples globally have an unmet need for contraception, and 80 million women have an unintended pregnancy [35]. Sub-Saharan Africa has the highest percentage of women with unmet reproductive health needs which is approximately one out of every four women [36]. A study of physical health experiences of women after birth and abortion of unwanted pregnancy showed that the safety of induced abortion compared to child birth and highlighted the risk of serious morbidity and mortality associated with childbirth [37]. A study by Jalay et al., showed that unintended pregnancies are more likely to result in adverse health behaviors [14].

Only a few studies have been conducted relating maternal health outcomes in developing countries. This study may aid in implementation new plan and policy to reduce maternal and child mortality since reducing one unintended pregnancy is equivalent to improving the health of both the mother and child. This study has some without limitation. First, our subject only included women who were currently pregnant at the time of survey, so the result from the experience of unintended pregnancy in women cannot be generalized to the entire population. Next, this is a cross sectional study that uses logistic regression analysis for statistical analysis therefore we cannot claim a cause–effect relationship between the unintended pregnancies and associated factors. Lastly, because of the small sample size classification based on education status, our study subjects are classified as either educated or uneducated.

## Conclusion

The unintended pregnancy rate in reproductive aged, pregnant women of the Kwango District, DRC was very high. This study explored some of the factors associated with unintended pregnancy. Household income level, education of the participants, age of the last child, past use of a FP method, ownership of a cell phone, and family size were significantly associated with an increased likelihood of unintended pregnancy. In conclusion, unintended pregnancy in an area with a high fertility rate and high mortality is a tremendous burden to both women and the country. Unintended pregnancy prevention and intervention should incorporate female education, women’s access to property and income, increased FP counseling and service utilization, child-birth spacing, and education on behavior changes. In conclusion, effective programs such as appropriate counseling should be provided for reproductive age women to increase awareness of unintended pregnancy.

## Abbreviations

AOR, Adjusted Odd Ratio; CI, Confidence Interval; DRC, Democratic Republic of the Congo; FP, Family Planning; KOICA, Korea International Cooperation Agency; OR, Odds Ratio
